# Integrated Transcriptome and Proteome Analysis Provides Insight into the Ribosome Inactivating Proteins in *Plukenetia volubilis* Seeds

**DOI:** 10.3390/ijms23179562

**Published:** 2022-08-24

**Authors:** Guo Liu, Zhihua Wu, Yan Peng, Xiuhua Shang, Liqiong Gao

**Affiliations:** Research Institute of Fast-Growing Trees, Chinese Academy of Forestry, 30 Mid Renmin Avenue, Zhanjiang 524022, China

**Keywords:** *Plukenetia volubilis*, transcriptome, proteomics, ribosome-inactivating proteins, physicochemical property, expression pattern

## Abstract

*Plukenetia volubilis* is a highly promising plant with high nutritional and economic values. In our previous studies, the expression levels of ricin encoded transcripts were the highest in the maturation stage of *P. volubilis* seeds. The present study investigated the transcriptome and proteome profiles of seeds at two developmental stages (Pv-1 and Pv-2) using RNA-Seq and iTRAQ technologies. A total of 53,224 unigenes and 6026 proteins were identified, with functional enrichment analyses, including GO, KEGG, and KOG annotations. At two development stages of *P. volubilis* seeds, 8815 unique differentially expressed genes (DEGs) and 4983 unique differentially abundant proteins (DAPs) were identified. Omics-based association analysis showed that ribosome-inactivating protein (RIP) transcripts had the highest expression and abundance levels in Pv-2, and those DEGs/DAPs of RIPs in the GO category were involved in hydrolase activity. Furthermore, 21 RIP genes and their corresponding amino acid sequences were obtained from libraries produced with transcriptome analysis. The analysis of physicochemical properties showed that 21 RIPs of *P. volubilis* contained ricin, the ricin_B_lectin domain, or RIP domains and could be divided into three subfamilies, with the largest number for type II RIPs. The expression patterns of 10 RIP genes indicated that they were mostly highly expressed in Pv-2 and 4 transcripts encoding ricin_B_like lectins had very low expression levels during the seed development of *P. volubilis*. This finding would represent valuable evidence for the safety of oil production from *P. volubilis* for human consumption. It is also notable that the expression level of the Unigene0030485 encoding type I RIP was the highest in roots, which would be related to the antiviral activity of RIPs. This study provides a comprehensive analysis of the physicochemical properties and expression patterns of RIPs in different organs of *P. volubilis* and lays a theoretical foundation for further research and utilization of RIPs in *P. volubilis*.

## 1. Introduction

Ribosome-inactivating proteins (RIPs) are toxic N-glycosidase enzymes found in most plant species and distributed across multiple organs, with the function of defense against fungal or viral infections [1,2]. Ricin toxin (RT), belonging to the RIP family of toxins, is a protein with highly toxic properties, present in abundance in the seeds of castor bean (*Ricinus communis*) [3,4,5]. The members of the RIP family are classified as type I RIPs, which include a single polypeptide chain (~30 kDa) with RNA-*N*-glycosidase enzyme activity, and type II RIPs that include a lectin domain (ricin toxin B_chain, RTB) and a glycosidase domain (ricin toxin A_chain, RTA) [6]. These two chains are connected by a single interchain disulfide bond [7]. Pokeweed antiviral protein (PAP) and trichosanthin (TCS) belong to the subfamily of type I RIP, but abrin and RT are typical type II RIPs [8]. Ricin has attracted interest mainly due to its cytotoxicity to mammalian cells and potential applications as biological weapons, which makes it to be considered a moderate threat by the US Center for Disease Control and Prevention (CDC) [6]; moreover, ricin also can be used as a research tool to study its intracellular transport and therapeutic effect on tumors [9,10] and AIDS (acquired immune deficiency syndrome) [10,11]. These applications are attributed to the toxicity of RT, which is a thousand times higher than that of arsenic and 2–3 times higher than that of cobra venom. Ricin is a protein with lectin domains, exhibiting hemagglutination activity [12].

*Plukenetia volubilis* Linneo (Euphorbiaceae) is an underutilized oilseed crop with high nutritional values, native to the Amazon basin of South America, and has been traditionally utilized by indigenous Incas since pre-Hispanic times [13,14]. The mature seeds contain approximately 35.0~60.0% lipids, of which more than 90.0% are polyunsaturated fatty acids (PUFAs). The PUFAs in the seeds of *P. volubilis* comprised approximately 35.2~50.8% α-linolenic acid (C18:3n-3, ω-3, ALA) and approximately 33.4~41.0% linoleic acid (C18:2n-6, ω-6, LA), which are the two fatty acids essential for humans and must be obtained from the diet [15]. The seeds also contain approximately 26.6~31.6% protein and have antioxidant properties due to the presence of phenols, carotenoids, and tocopherols [16]. Due to the excellent nutritional composition in its seeds, especially lipid, *P. volubils* has gained increasing popularity and awareness in the global markets in recent years [17]. Although cold-pressed edible oil from *P. volubilis* seeds has been shown to not contain toxins or harmful substances for health and thus is safe for human consumption, the roasted seeds from *P. volubilis* are not approved by the European Union (EU) due to a lack of relevant knowledge about the composition and content of alkaloids in these seeds [18]. Sruchamnong et al. [19] found that the fresh leaves and seeds of *P. volubilis* contained alkaloids, saponins, and possibly lectins, the main groups of naturally occurring plant toxins, which belong to the main classes of secondary metabolites and can be found in many parts of plants, including seeds, bark, leaves, and roots. These phytotoxins might become unstable under heat treatment, suggesting that the long-term consumption of large quantities of fresh seeds and leaves of *P. volubilis* should be avoided [18,19]. In addition, Liu et al. [20] and Wang et al. [21] performed the transcriptome analysis of five and two developmental stages of *P. volubilis* seeds, respectively. Surprisingly, the amounts of RIPs transcripts were the highest at the maturity stage or the fast oil accumulation stage, and the expression levels of those transcripts were greater than 10^6^. Specifically, in the seeds of *R. communis* seeds, 10 RIP genes, including 4 type I and 6 type II, also showed the highest expression levels in stages approaching maturity [22]. Therefore, knowledge of the physicochemical properties and expression patterns of RIP genes in *P. volubilis* is important in attempts to ensure a sustained supply of polyunsaturated fatty acids (PUFAs) without posing any threat to human health.

Considering the current global challenges, it is absolutely imperative to ensure food security, mitigate climate change, and alleviate malnutrition. Therefore, underutilized crops may help improve agricultural resilience, eliminate the need for external inputs, build climate resilience, facilitate dietary diversification, and improve income opportunities for farmers [14]. *P. volubilis* is a promising new crop in this regard with great potential for further domestication, and their seed oil has an excellent composition and good sensory acceptability [16]. The planting processes and requirements of *P. volubilis* are relatively simple, so it is a well-established plant that has numerous potential applications in gastronomy, cosmetics, and medicine.

According to the dynamic change of the fatty acids in the development of *P. volubilis* seeds [23] and the expression trend of RIP genes in the transcriptome of five and two developmental stages [20,21], a combination of transcriptomic and proteomics were used to identify differentially expressed genes (DEGs) and differentially abundant proteins (DAPs) in two developmental stages of *P. volubilis* seeds in the present study. Subsequently, RIP genes, physicochemical properties of ribosome-inactivating proteins, and expression patterns of RIP genes in *P. volubilies* were explored. The construction of transcriptional regulatory networks would help understand the gene functions. These results will lay a theoretical foundation for further utilization of RIPs in *P. volubilis*.

## 2. Results

### 2.1. Sequencing, Assembly, and Annotation

To determine changes in gene expression across the transcriptome at two developmental stages of *P. volubilis* seeds, RNA sequencing analysis was performed using RNA samples including Pv-1 (10 days after pollination (DAP)) and Pv-2 (100 DAP). A total of 227.63 million reads were generated from the two libraries (an average of 37.94 million), encompassing 34.14 Gb of sequence data (an average of 5.69 Gb). After stringent quality assessment and data filtering, a total of 227.48 million high-quality reads (an average of 37.91 million) were selected for further analysis. The Q20 percentage reached more than 98.29%, and the GC content ranged from 44.36% to 45.71%. The de novo transcriptome assembly was carried out using the Trinity software (2.8.6). As a result, 54.25 M bases were assembled, and 53,224 unigenes were obtained, with an average length of 1019 bp and an N50 of 1834 bp. Results of the BUSCO analysis showed that by the assembly, 91.60% of the conserved single-copy orthologous genes, including 77.01% complete (C) and 14.58% fragment (F) genes, were retrieved. The high percentage of completeness (C) and low percentages of both fragments (F) and missing sequences (M) (8.40%) indicated that the transcriptome assembly had a good representation of the transcriptome of *P. volubilis* seeds.

Using ESTScan 2.0b software with a cut-off E-value of 1 × 10^−^^5^, a total of 40,172 unigenes were annotated (Figure S1a), of which 38,331 (72.02%), 20,497 (51.02%), 30,859 (57.98%), 24,366 (45.78%), and 36,410 (68.41%) unigenes showed significant similarities to known genes in the NR, GO, SwissProt, KOG, and KEGG databases, respectively. Approximately 34.71% (13,942) of unigenes could be assigned to a homolog in all five databases (Figure S1a). As shown in Figure S1b, a large number of unigenes in *P. volubilis* showed close identities to the genes in other plant species. The highest number of homologous genes in *P. volubilis* (13,982 unigenes, 36.48% of the total) was identified in *R. communis*, followed by *Brassica napus* (3898, 10.17%), and *Jatropha curcas* (3130, 8.17%).

According to the gene ontology (GO) annotation (Figure S2), the sub-categories metabolic process (GO:0008152, 13,208 unigenes, 64.44%) and cellular process (GO:0009987, 11,466 unigenes, 55.94%) were most prominently represented in the category of biological process (BP). For the category of molecular function (MF), catalytic activity (GO:0003824, 10,762 unigenes, 52.51%) and binding (GO:0005488, 9075 unigenes, 44.27%) were the most highly represented GO terms. For the cellular component (CC) category, however, the most frequently represented terms were cell (GO:0005623, 8939 unigenes, 43.61%) and cell part (GO:0044464, 8935 unigenes, 43.60%). The KOG annotation results (Figure S3) showed that the largest number of unigenes was clustered into the functional category of “general function prediction only” (5010 unigenes, 20.56%), followed by “post-translational modification”, “protein turnover”, “chaperones” (3273 unigenes, 13.43%), and “signal transduction mechanisms” (2971 unigenes, 12.19%). Around 1033 (4.24%) unigenes were assigned to the cluster of “lipid transport and metabolism”. Based on the KEGG pathway assignment, 30,859 unigenes were assigned to a total of 133 KEGG pathways (Table S1), with the majority of these unigenes involved in the pathways of metabolic pathways (ko01100, 4404 unigenes, 12.10%) and biosynthesis of secondary metabolites (ko01110, 2461 unigenes, 6.76%). Among the 14 KEGG pathways associated with lipid metabolism, 161 unigenes were mapped to the pathways of the glycerolipid metabolism (ko00561), followed by glycerophospholipid metabolism (ko00564; 159 unigenes) and fatty acid degradation (ko00071; 132 unigenes).

### 2.2. Enrichment Analysis of DEGs Based on GO and KEGG Pathways

We identified mRNAs with a fold change ≥2 and a false discovery rate (FDR) < 0.05 as significant DEGs. In total, a set of 8815 DEGs (2188 upregulated and 6627 downregulated) showed ≥2-fold changes or different gene expression levels between the two libraries. Of 18 DEGs with RPKM values > 1000 in the Pv-1 (Table S2), 3 DEGs encode a proline-rich protein that is a structural constituent of cell wall proteins (Unigene0006989, Unigene0007377, and Unigene0009962), and 2 DEGs encode chlorophyll a/b-binding protein that may be involved in pigment biosynthesis or the assembly of thylakoid membranes (Unigene0000529 and Unigene0002244). It is noteworthy that among 27 DEGs with RPKM values > 1000 in the Pv-2 (Table S3), the 4 most abundant transcripts (Unigene0000208, Unigene0000207, Unigene0000209, and Unigene0000172) and Unigene0012386 code for ricin-like protein and the ribosome-inactivating protein gelonin, respectively, indicating the toxicity of raw seeds of *P. volubilis*. Transcripts encoding 2S albumin-like (Unigene0003223, Unigene0049069, Unigene0046600, Unigene0011183, and Unigene0048736), legumin-like (Unigene0048787, Unigene0010858, and Unigene0010856) and vicilin-like proteins (Unigene0049803 and Unigene0044374) were also abundant; these proteins are specifically synthesized during seed maturation. Other abundant transcripts of the *Oleosin* genes (Unigene0044871, Unigene0028565, and Unigene0050906) are highly abundant in land plants and could positively regulate total seed oil accumulation [24,25].

**Figure 1:**
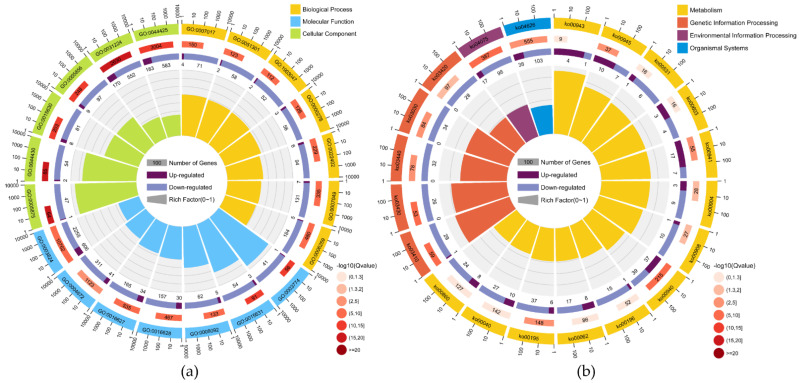
Top 20 of GO function (**a**) and KEGG pathway (**b**) enrichment of DEGs in the transcriptome of two developmental stages of *P. volubilis* seeds. The first circle: top 20 GO terms/KEGG pathways. The number outside of the circle is the coordinate ruler of the gene number. Different colors represent different GO terms/KEGG A classes; the second circle: the number and Q value of the GO term/KEGG pathway in the background gene. The more genes, the longer the bars, and the smaller the *Q*-value, the redder the color; the *Q*-value is the *p* value corrected by the FDR method. The third circle: a bar chart of the proportion of up and down-regulated genes, dark purple represents the proportion of up-regulated genes, and light purple represents the proportion of down-regulated genes; the specific values are shown below. Fourth circle: RichFactor value of each KEGG pathway.

GO enrichment analysis was carried out, and based on primary biological functions, DEGs were classified into three main categories, including BP (1476 terms; 3987 unigenes), MF (224 terms; 3878 unigenes), and CC (574 terms; 2336 unigenes) (Table S4). Among the top 20 GO enrichment terms (Figure 1a), catalytic activity (GO:0003824) contained the largest number of unigenes (2861; 606/2255, up-/downregulated, respectively), followed by membrane part (GO:0044425; 766; 183/583, up-/downregulated, respectively), and intrinsic component of membrane (GO:0031224; 722; 170/552, up-/downregulated, respectively). In particular, three unigenes (Unigene0000207, Unigene0000208, and Unigene0000209) encode a ricin-like protein and one unigene encodes the ribosome-inactivating protein gelonin (Unigene0012388) with extremely high expression levels at the Pv-2 stage were enriched in hydrolase activity (GO:0016787).

To further determine whether the metabolic pathways were involved during the two developmental stages of seeds, 129 KEGG pathways were represented by DEGs (1928 unigenes) (Table S5). The pathway represented by the largest number of unigenes (749) was the metabolic pathway (Ko01100). The top 20 enriched KEGG pathways are shown in Figure 1b, among which pathways of Photosynthesis (ko00195), Photosynthesis-antenna proteins (ko00196), and Porphyrin and chlorophyll metabolism (ko00860) are closely related to plant photosynthesis.

### 2.3. Protein Identification and Functional Annotation

**Figure 2:**
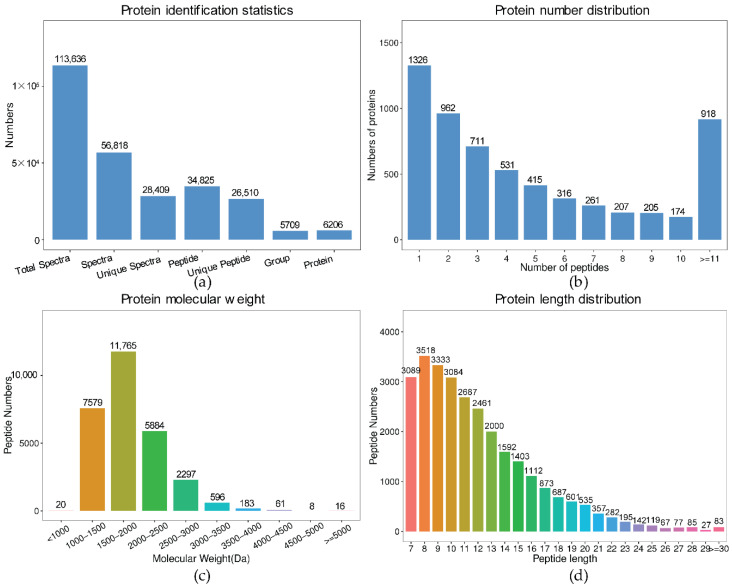
(**a**) The identification results of proteins in *P. volubilis* seeds; (**b**) Number distribution of all identified unique proteins. The abscissa is the number of peptides, and the ordinate is the number of proteins in this peptide number; (**c**) Molecular weight (Da) of all identified proteins; (**d**) Length distribution of all identified protein.

The comparative proteome analysis of Pv-1 and Pv-2 was carried out by iTRAQ technology for the comprehensive transcriptome study. A total of 113,636 total spectra, 56,818 spectra, 28,409 unique spectra, 34,825 peptides, 26,510 unique peptides, and 6026 proteins were identified (Figure 2a). The analysis of the characteristics of peptides showed that 4700 (78.00%) peptides were related to at least 2 unique peptides (Figure 2b). The mass of peptides ranged from 1000 to 4000 Da, and the peptides within the range of 1000–3000 Da accounted for 96.89% (27,525) of total peptides (Figure 2c). The average length of the identified peptides was 11.99 amino acids, which was within the reasonable range (Figure 2d).

The changes in the structure of protein species and average masses in seeds of *P. volubilis* at two growth stages can be used to study protein function. Out of a total of 6026 identified proteins, 4336 (71.95%), 2613 (43.36%), and 4534 (75.24%) proteins could be annotated in GO, KEGG, and KOG databases, respectively (Figure S4). Altogether, 5375 (89.20%) proteins were successfully annotated in these three public databases. Based on GO categorization, 4336 proteins were classified into a total of 2348 GO terms, including BP (1536 terms), CC (245 terms), and MF (567 terms) (Table S6). In the BP category, metabolic process (GO:0008152) and cellular process (GO:0009987) were the predominant terms, followed by organic substance metabolic process (GO:0071704). In the CC category, cell (GO:0005623) and cell part (GO:0044464) were the predominant terms, followed by intracellular (GO:0005622) and intracellular part (GO:0044424). In the MF category, however, catalytic activity (GO:0003824) and binding (GO:0005488) were the main distributed terms, followed by organic cyclic compound binding (GO:0097159) and heterocyclic compound binding (GO:1901363). A total of 2613 proteins were assigned to 128 pathways in the KEGG database (Table S7). The most represented pathways included the metabolic pathway (ko01100, 1173 proteins), secondary metabolite biosynthesis (ko01110, 645 proteins), and ribosome (ko03010, 257 proteins). Notably, 14 pathways, such as fatty acid degradation (ko00071), glycerolipid metabolism (ko00561), fatty acid biosynthesis (ko00061, 35 proteins), etc., were closely linked to lipid biosynthesis and metabolism and took place during the seed germination of *P. volubilis*. In total, 4534 proteins were assigned to COG classes (Figure S5). Among the 25 COG categories, “General function prediction only” represented the largest group (763 proteins), followed by “Post-translational modification”, “protein turnover”, and “chaperones” (712 proteins). The “Lipid transport and metabolism” category contained 221 proteins.

### 2.4. Enrichment Analysis of DAPs Based on GO and KEGG Pathways

A total of 4983 proteins were identified as DAPs at two developmental stages of *P. volubilis* seeds (fold change > 1.2, *p*-value < 0.05), of which 169 DAPs were upregulated and 4814 DAPs were downregulated (Table S8). Consistent with the RNA-seq data, the number of upregulated DAPs was lower than that of downregulated DAPs. It is particularly noteworthy that based on the relative quantification, the number of ricin-like proteins (Unigene0000207 and Unigene0000209) was the highest in Pv-2, followed by legumin proteins (Unigene0048787, Unigene0010856, and Unigene0010858). However, the quantity of β-glucosidase (Unigene0018636) in the Pv-1 was the highest, followed by the MLP-like protein (Unigene0051229), and ricin (Unigene0000207). The proteins closely involved in fatty acid biosynthesis, such as oil body-associated proteins (Unigene0048411 and Unigene0032502), Oleosins (Unigene0028565 and Unigene0050906), FAD3 (Unigene0043398), FAD2 (Unigene0034350), SAD (Unigene0011486), and long-chain Acyl-CoA synthetase (Unigene0026569), were all significantly up-regulated in the fast oil accumulation stage.

GO functional annotation and KEGG pathway enrichment analysis were used to compare the changes in annotated DAPs. A total of 3619 DAPs were enriched in 2256 GO terms, including 1483 terms in BP, 234 terms in CC, and 539 terms in MF (Table S9). For the BP category, DAPs were mainly related to the metabolic process (GO:0008152, 2476 DAPs), the cellular process (GO:0009987, 2021 DAPs), and the organic substance metabolic process (GO:0071704, 1881 DAPs). In the CC category, DAPs were mainly associated with the cell (GO:0005623), cell part (GO:0044464), intracellular (GO:0005622), and intracellular part (GO:0044424). In the MF category, however, DAPs were mainly associated with catalytic activity (GO:0003824), binding (GO:0005488), organic cyclic compound binding (GO:0097159), and heterocyclic compound binding (GO:1901363). The KEGG enrichment analysis results showed that DAPs were assigned to 127 pathways, of which 96 were metabolic pathways (Table S10). In enrichment of the top 20 pathways based on *Q*-value (Figure S6), the most enriched pathways included Spliceosome (ko03040), Plant-pathogen interaction (ko04626), and Ascorbate and aldarate metabolism (ko00053). A total of 23 and 35 DAPs were enriched in the phosphatidylinositol signaling system (ko04070) and the plant MAPK signaling pathway (ko04016), respectively, among the first 20 pathways, which were related to signal transduction [26,27].

### 2.5. Association Analysis of Transcriptome and Proteome Data

**Figure 3:**
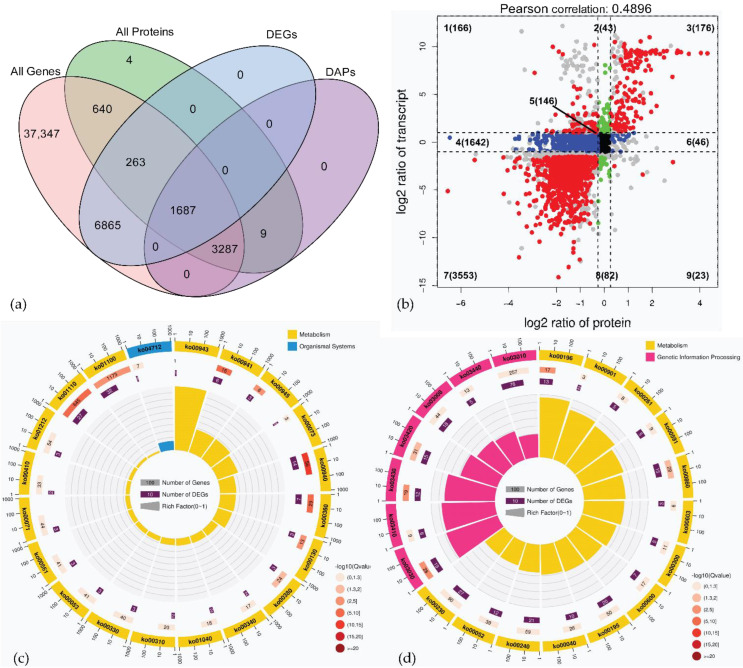
(**a**) Venn diagram of DEGs in all genes and DAPs in all proteins; the description of what is contained in the first panel; (**b**) scatter plot of nine-quadrant associate analyses of transcripts and proteins in Pv-1 and Pv-2. Each dot represents a gene/protein. The dashed line on the abscissa represents the FC threshold of DEGs (FC ≥ 2.0), the dashed line on the ordinate represents the FC threshold of DAPs (FC ≥ 1.2), the genes/proteins outside the threshold line are DEGs/DAPs, and the genes/proteins inside the threshold line are not DEGs/DAPs. Numbers 1–9 represent each quadrant, and the number of points in each quadrant showed in parentheses. Quadrants 1, 2, and 4 indicate that the protein abundance was lower than the RNA expression. In 3 and 7, the RNAs correspond with the related proteins. Quadrant 5 represents that the proteins and transcripts were commonly expressed with no difference. Quadrants 6, 8, and 9 indicate that the protein abundance was higher than the RNA expression. (If the FC is reached and the *p* value is not reached, it will be shown as the gray point). Top 20 of KEGG pathway enrichment of DEGs/DAPs in quadrant 3 (**c**) and quadrant 7 (**d**). The first circle: numbers outside of the circle are the coordinate rulers of the gene number, different colors represent different KEGG A classes; the second circle: the number and *Q*-value of the KEGG pathway in the background gene. The more genes, the longer the bars, and the smaller the *Q*-value, the redder the color; the *Q*-value is the *p*-value corrected by the FDR method. The third circle: a bar chart of the proportion of up and down-regulated genes, dark purple represents the proportion of up-regulated genes, and light purple represents the proportion of down-regulated genes; the specific values are shown below. Fourth circle: RichFactor value of each KEGG pathway.

A global correlation analysis was performed between the proteins and their corresponding transcripts to explore the consistency between the transcriptome and proteome data [28]. Association analysis revealed that 5877 proteins matched the transcripts, of which 4974 were DAPs, 1950 were DEGs, and 1687 were both DAPs and DEGs (Figure 3a). Similar to a previous study on *Drosophila melanogaster*, only a moderate correlation (R = 0.49) was observed between transcriptome and proteome data.

Quantitative and enrichment analyses of genes and proteins were performed in each region of the nine-quadrant map, which was drawn based on changes in the expression of genes and proteins at the transcriptome and proteome levels. As shown in Figure 3b, the transcripts/proteins (146) concentrated at the center of the plot (quadrant 5) were NDEGs/NDAPs, respectively. The proteins in quadrant 4 (1443) and quadrant 6 (17) were DAPs whose corresponding genes were not differentially expressed. The proteins in quadrant 2 (41) and quadrant 8 (28), however, were not differentially expressed, but their corresponding genes were differentially expressed. The proteins in quadrants 1 (63) and 9 (3) had the opposite expression patterns from their transcripts, while the proteins in quadrants 3 (141) and 7 (1480) showed the same expression patterns as their transcripts. This indicated that the expression of proteins in quadrants 3 and 7 was regulated at the transcriptional level. However, for proteins in quadrants 1, 2, 4, 6, 8, and 9, there were regulatory events at the post-transcriptional or translational levels (e.g., miRNAs regulate the translation of target genes and inhibit the expression of proteins) [29], and therefore, the gene expression cannot fully represent the abundance of these proteins.

The correlation analysis between the transcriptome and proteome data in GO function and KEGG pathway was performed to compare the gene function and metabolic pathway in terms of their similarities and differences between the two groups. GO analysis of the DEGs/DAPs upregulated in the fast oil accumulation stages of *P. volubilis* seeds (Table S11) revealed that the vacuole (GO:0005773) and lipid particle (GO:0005811) were enriched in the CC category, suggesting that oil body formation and development took place in Pv-2. Remarkably, based on GO enrichment analysis, the GO terms, including enzyme inhibitor activity (GO:0004857) and catalytic activity (GO:0003824) were enriched in the MF category; and the negative regulation of catalytic activity (GO:0043086), regulation of hydrolase activity (GO:0051336), negative regulation of hydrolase activity (GO:0051346), negative regulation of peptidase activity (GO:0010466), regulation of peptidase activity (GO:0052547), regulation of proteolysis (GO:0030162), and negative regulation of proteolysis (GO:0045861) in the BP category were all significantly enriched. This indicated that the activity of proteins decreased during seed ripening. Particularly, 5 ricin-like DEGs/DAPs including Unigene0000207, Unigene0000208, Unigene0000209, Unigene0012388, and Unigene0037089 were involved in hydrolase activity (GO:0016787), and one RIP (Unigene0012386) was also involved in hydrolase activity, acting on glycosyl bonds (GO:0016798).

KEGG pathway enrichment analysis showed that DEGs/DAPs were mainly involved in metabolism pathways (ko01100, 749 DEGs/963 DAPs), followed by biosynthesis of secondary metabolites (ko01110, 435 DEGs/515 DAPs), and ribosome (ko03010, 185 DEGs/213 DAPs) (Figure S7). In quadrant 3, 141 unigenes showed a positive relationship between mRNA enrichment and protein abundances. The upregulated DEG/DAP pairs were mainly enriched in metabolic pathways (ko01100), biosynthesis of secondary metabolites (ko01110), and phenylpropanoid biosynthesis (ko00940). Among the top 20 pathways in quadrant 3, five pathways related to lipid metabolism were enriched, and the fatty acid degradation pathway (ko00071) and glycerolipid metabolism pathway (ko00561) were also significantly enriched (Figure 3c). In quadrant 7, in Pv-2, 1480 downregulated DEGs/DAPs were involved in significantly enriched pathways of (*Q*-value < 0.05) ribosome (ko03010), spliceosome (ko03040), RNA transport (ko03013), and DNA replication (ko03030) (Figure 3d), which were growth-related and developmental pathways, and key enzymes were significantly downregulated in Pv-2. Plant-pathogen interaction (ko04626) and phosphatidylinositol signaling system (ko04070) were enriched in 51 and 20 DAPs, respectively, in quadrant 7.

### 2.6. Analysis of Physicochemical Properties of RIPs

Analysis of spatial protein structure is of great significance for understanding the function and implementation of proteins, the interaction between biological macromolecules, and the development of medicine and pharmacy [30].

Based on the transcriptomic data, 21 transcripts from RIP-seq experiments were screened, but only 8 proteins were identified based on the proteomic data. Using the DNA sequences of transcripts, we translated the encoded proteins of 21 RIP genes, and their bioinformatics analysis was performed (Table S12). The analysis of ExPASyParam showed that the length of different identified transcripts was variable, ranging from 67 to 559 amino acids. The protein encoding Unigene0004179 had the largest number of amino acids (559), followed by Unigene0052941 (552), while the proteins encoding Unigene0012387, Unigene0012388, and Unigene0000208 had the lowest number of amino acids. This may be due to the presence of variable shear strength. The theoretical isoelectric points (PI) of 21 RIPs ranged from 4.43 to 9.78, with the highest PI for the unigene0000786 and the lowest PI for unigene0030485. According to the PI values of these 21 RIPs, RIPs in *P. volubilis* had slightly higher contents of proteins, which was attributable to the PI values of 12 RIPs that were less than 7. The instability indices (II) of 21 RIPs in *P. volubilis* ranged from 11.11 to 40.28 and were all classified as stable proteins except unigene0018529 (II = 40.28). Based on aliphatic indices of 21 RIPs, the number of aliphatic amino acids in unigene0005315 was the highest (123.13), followed by unigene0012388 and unigene0018530, while the number of aliphatic amino acids in unigene0043472 protein was the lowest (64.64). Among them, 13 RIPs were hydrophilic, but 8 were hydrophobic. This indicates that there were some differences in physicochemical properties of different RIPs in *P. volubilis*.

The Batch CD-search tool, Pfam, and SMART Online software programs were used to search the protein conservative domains. The results showed that 10 transcripts contained the conservative domain ricin with (QxW)_n_ motif (cl23784, IPR001574), while 9 transcripts contained the RIP superfamily domain with Yx_n_Yx_n_ExxRx_n_W motif (cl08249, IPR017989), and 4 transcripts contained the ricin B-like lectin domain with (QxW)_3_ motif (cl40832, IPR040249). The motif of Yx_n_Yx_n_ExxRx_n_W plays a role in stabilizing the active center of the enzyme, and the QxW domain has been found to be associated with diverse functions such as enzyme activity, inhibition of toxicity, and signal transduction [31]. Among 21 RIPs, 15 contained the transcripts encoding the signal peptide. The modification of proteins through glycosylation not only affects the biological activity, spatial conception, and localization and transport of proteins but also plays an important role in specific biological processes such as cell communication, molecular recognition, signal transduction, etc., [32,33]. In the current study, the NetOGlyc software was used to analyze the glycosylation sites. There were 1~8 O-glycosylation sites in 12 RIPs. Unigene0018947 had 8 O-glycosylation sites, whereas Unigene0000209 had 6 O-glycosylation sites. Protein phosphorylation refers to the process of transferring the phosphate group from an ATP to amino acid residues (serine, threonine, and tyrosine) within substrate proteins catalyzed by protein kinases or binding GTP under the action of signals (usually GTP replaces GDP) [34]. The analysis using the NetPhos software showed that all 21 RIPs had phosphorylation sites (8~94), of which 94 were on the Unigene0052941, while 70 were on Unigene0004179. Consistent with the results of Rezaei-Moshaei et al. [35], the number of potential Ser and Ther phosphorylation sites were much higher than Tyr phosphorylation sites (Table S12). Furin serves as an endoproteinase in eukaryotes. It recognizes the specific amino acid sequence and after two self-cleavages in the endoplasmic reticulum in the Golgi body, it cleaves many crucial precursors of peptides and proteins in the secretory pathway, facilitating the bioactivity of precursors [33]. Based on the prediction of the presence and location of furin cleavage sites by the ProP software, there was only one furin cleavage site in four RIPs. The results of the prediction of the regions of transmembrane proteins based on the HMM method showed that 5 transcripts in RIPs contained a transmembrane helix, and transmembrane proteins were expressed as i12–34o, i7–24o, o4–26i, i5–22o, and i5–27o. The prediction of protein subcellular localization using WoLF PSORT showed that three transcripts encoding a ricin-like protein, Unigene0004179 encoding ricin B-like lectin, and Unigene0018529 encoding type I RIP might be distributed to extracellular vesicles; two transcripts encoding type I RIP might be located in the cytoplasm; Unigene0005315 encoding ricin-like, three transcripts encoding ricin B-like lectin, and two transcripts encoding type I RIP might be distributed along cytoskeletal filaments; four transcripts encoding a ricin-like protein and four transcripts encoding type I RIP might be found in the chloroplast.

**Figure 4:**
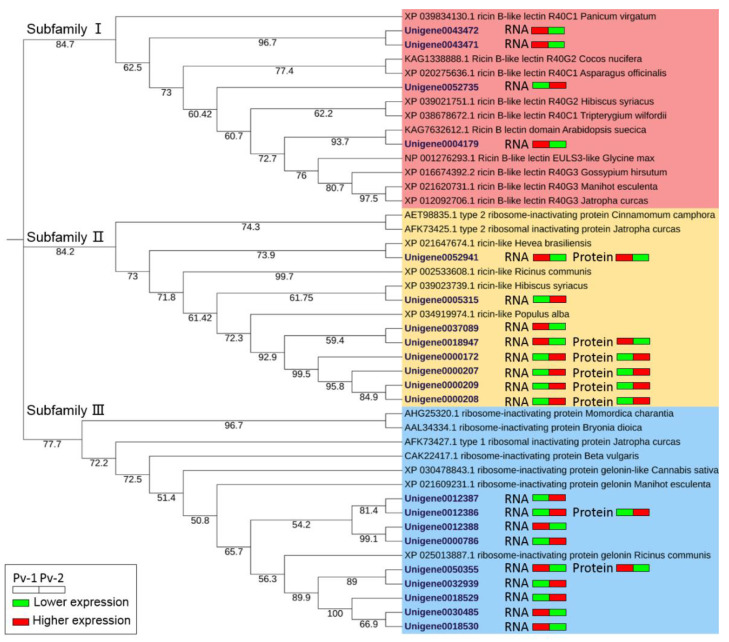
ML phylogenetic tree of 21 RIPs in *P. volubilis* and 23 RIPs in other plants with 1000 bootstrap replicates. The number below the branches indicates bootstrap percentages. The icons right to each RIP ID showed the expression patterns of each transcript/protein through the two seed developmental stages, with the green color indicating lower expression/abundance, while the red color indicates higher expression/abundance.

According to the results of the maximum likelihood estimation of phylogenetic tree construction, the RIPs in *P. volubilis* and other plants were divided into three subfamilies (Figure 4), similar to the RIPs in *R. communis*. Subfamily I was mainly composed of ricin B-like lectin proteins, while subfamily II contained the ricin-like proteins, and subfamily III consisted of type I RIPs. The differentiation of type I RIP (subfamily III) occurred early; however, the ricin B-like lectin (subfamily I) and the ricin-like protein (subfamily II) were differentiated relatively recently, with the differentiation of ricin B-like lectin occurring a little later than that of the ricin-like protein. Therefore, it was inferred that ricin B-like lectin that belongs to type II RIP could possibly have evolved from type I RIP. This inference was consistent with Li and Liu [31].

Based on the relative expressions of 21 RIP genes and the abundance of 8 RIPs in seeds at two different developmental stages from transcriptome and proteome data, 11 RIP genes were up-regulated at Pv-2 (the fast oil accumulation stage), whereas 10 RIP genes were up-regulated at Pv-1 (the initial stage). The trend of the accumulation of eight proteins was exactly consistent with the expression patterns of their corresponding genes. Subfamily II consisted of type 2 RIPs and ricin-like proteins, with the latter having the probability of being RTA. Six proteins in P. volubilis in the subfamily II were DAPs, four of which (Unigene0000172, Unigene0000207, Unigene0000208, and Unigene0000209) had the highest abundance at Pv-2. Additionally, the type I RIP (Unigene0012386) was also a DAP and abundantly accumulated at Pv-2.

### 2.7. The Validation of RT-qPCR Assay and Expression Patterns of RIP Genes in P. volubilis Seeds

**Figure 5:**
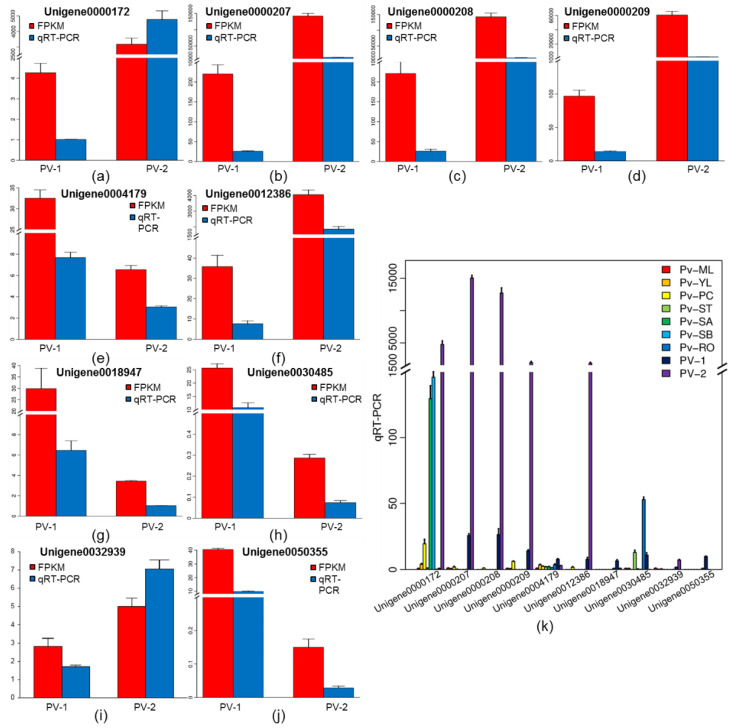
(**a**–**j**) Verification of the consistency of the RNA-Seq data using quantitative real-time PCR (qRT-PCR). (**k**) The expression patterns of the description of ten RIP genes in nine organs of *P. volubilis*. Pv-ML, mature leaf; Pv-YL, young leaf; Pv-PC, pericarp; Pv-ST, stem; Pv-SA, stem apex; Pv-SB, stem bark; Pv-RO, root young leaves. Error bars ± SD from 3 biologicals. The data were subjected to Student’s *t*-test.

To verify the accuracy of transcriptome data, 10 mRNAs with higher expression levels (greater than 50 in at least one sample) and relatively high read counts (usually greater than 20) in both groups were selected for RT-qPCR analysis (Figure 5a–j). The results showed that the relative expression trends of 10 genes by RT-qPCR were consistent with the transcriptome sequencing results. Meanwhile, a highly significant correlation (R^2 =^ 0.95) was found between RT-qPCR and RNA-seq data for these 10 RIP genes (Figure S8), which indicated that the transcriptome data were highly reliable.

To further identify the RIP genes in *P. volubilis* seeds, the expression levels of 10 genes with higher RPKM values (RPKM ≥ 10) were determined using RT-qPCR in nine organs in *P. volubilis*. Although 10 RIP genes were all highly expressed in *P. volubilis* seeds, individual RIP genes were differentially expressed in different organs (Figure 5k). Among these 10 genes, Unigene0000172 (the transcript of ricin-like protein) had the highest expression level in Pv-2 (the fast oil accumulation stage), which was 32.73 times and 36.83 times higher than in SB and SA, respectively. Similarly, the expression levels of three transcripts (Unigene0000207, Unigene0000208, and Unigene0000209) encoding ricin-like proteins and two transcripts (Unigene0012386 and Unigene0032939) encoding type I RIP also had the highest expression levels in Pv-2, around 586.51, 482.62, 140.69, 242.85, and 4.16 times higher than in Pv-1, respectively. Furthermore, Unigene0004179 encoding ricin B-like lectin and Unigene0050355 encoding type I RIP had the highest expression levels at Pv-1. Unigene00004179 specifically had a very low expression level in all organs, and Unigene0030485 encoding type I RIP was expressed higher at Pv-RO, indicating that most ricin genes were prominently expressed in mature seeds of *P. volubilis*.

### 2.8. Transcriptional Regulatory Network

**Figure 6:**
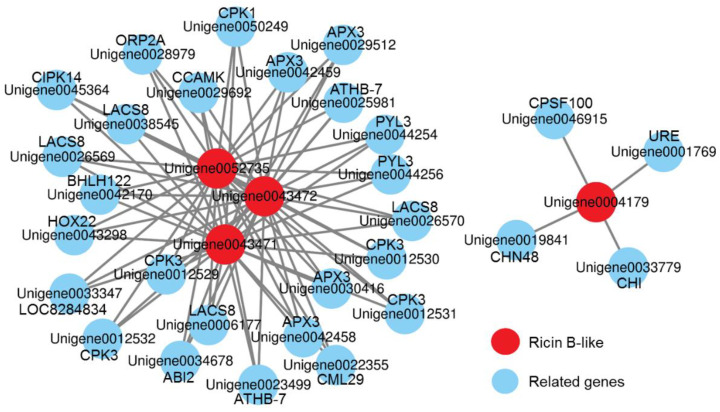
Transcriptional regulatory network of RIP genes in *P. volubilis* seeds.

Gene function is often closely related to the transcriptional regulation [36,37]. We analyzed 21 transcripts encoding RIPs and found that only 4 unigenes (Unigene0004179, Unigene0043471, Unigene0043472, and Unigene0052735) encoding ricin B-like proteins had paired-end relationships with the other 29 genes (Figure 6). The “combined score” indicated the strength of data support, and 29 unigenes were screened out when the threshold was set to 0.4. Among these 29 unigenes, 5 unigenes (Unigene0012529, Unigene0012530, Unigene0012531, Unigene0012532, and Unigene0050249) encoding calcium-dependent protein kinases (CPKs), 4 unigenes (Unigene0006177, Unigene0026569, Unigene0026570, and Unigene0038545) encoding long-chain acyl-CoA synthetase 8 (LACS8), and 4 genes encoding ascorbate peroxidase 3 (APX3) represented potential targets for 3 ricin B-like genes (Unigene0043471, Unigene0043472, and Unigene0052735). Notably, 4 ricin B-like genes had low expression levels during seed development, and thus, they could not be regarded as potential targets.

## 3. Discussion

*P. volubilis* is a highly promising crop, primarily due to the excellent nutritional composition of its seeds [13]. In the current study, a combination of transcriptomic and proteomic approaches was used to analyze *P. volubilis* seeds at two developmental stages (the initial stage and the fast oil accumulation stage). The association analysis for two-omics data was then performed. We obtained a total of 227.61 million 150 bp paired-end reads from two libraries (an average of 37.94 million), which were higher in number than those obtained by Wang et al. [21], who reported 52.6 million 90 bp paired-end reads from two libraries, with equal division of reads between the two (26.3 million for each). Transcriptome analysis at two developmental stages of *P. volubilis* seeds revealed the presence of a total of 53,224 unigenes, and the expression of 8815 unigenes differed, with at least a two-fold change between the two libraries. Compared with the transcriptome of five developmental stages of seeds [20], the gene number (53,224) obtained in this study was higher than 44,797 in the transcriptome of five developmental stages, and the N50 (1834), average length (1019), total assembled bases (54.25 M) were rather fewer than other data (2299, 1345, 60.67 M, respectively) in the transcriptome of five developmental stages. This may be associated with the upgrade of data filtering. From the annotation of GO, KOG, and KEGG, the transcriptome of five developmental stages of *P. volubilis* seeds [20] was essentially in agreement with the results of this study. A total of 6026 proteins and 4983 DAPs were identified by proteomic analysis. It is a very common protein post-translational modification (PTM) in organisms that plays an important role in the process of signal transduction at the single-cell level [38], and the association analysis revealed that transcriptome abundance and protein levels were only moderately correlated. This may be due to the effects of translation regulation, PTM, and the rate of protein transcription [37]. As the next step, we can consider other post-transcriptional regulatory binding sites as the important regulatory targets for the development of *P. volubilis* seeds.

The publication of the caster bean genome revealed the presence of 28 genes in the ricin gene family [39]. In our study, 21 transcripts and 8 proteins were identified in the transcriptome of *P. volubilis* seeds. The length of the different transcripts identified was variable, and some smaller genes (Unigene0012387 and Unigene0012388) could be nonfunctional or pseudogenes; furthermore, start and stop codons could be predicted, making it difficult to determine whether the genes were functional or not. Bioinformatics methods were employed to analyze the nucleotide and amino acids homology between sequences of 21 RIP genes, their possible protein domains, and the topology of transmembrane proteins. The possible protein domains were ricin, ricin-B-lectin, or RIP domains. There were phosphorylation sites in all 21 RIPs and more Ser and Ther phosphorylation sites than Tyr phosphorylation sites. It has been shown that the functional effects of protein phosphorylation are site-dependent, and phosphorylation occurs at a specific site [35]. Among the 21 RIPs, 15 RIPs were signal peptides, and O-glycosylation sites were found in 11 RIPs. Four RIPs were found to have furin cleavage sites, and five had a transmembrane domain. A total of 21 RIPs were distributed in the chloroplast and cytoskeleton and extracellular and cytoplasmic regions in the seeds of *P. volubilis*. Among them, five RIPs with the highest levels of expression and highest abundance levels in Pv-2 were most likely located in extracellular regions or chloroplast. The phylogenetic tree of the sequences of 21 RIPs in *P. volubilis* and other RIPs in other plants revealed that the RIPs in *P. volubilis* were divided into three subfamilies. The clustering results were consistent with the previous study of Loss-Morais [22] and provided new evidence for the hypothesis that type II RIPs evolved from type I RIPs. Of these three subfamilies, the differentiation of subfamily I (ricin B-like lectin) occurred late, which could be due to the fact that the ricin B chain is a product of gene duplication [40,41]. Similar to the RIPs in *R. communis*, the number of type II RIPs was the largest, with the highest abundance and expression levels. It is worth noting that the four transcripts encoding ricin-like proteins and Unigene0012386 encoding type I RIPs had the highest expression levels in Pv-2, but the four transcripts encoding ricin B-like lectin protein had relatively low expression levels during seed development of *P. volubilis*. Type II RIPs were highly toxic on RTB, which promotes the entry of RTA into host cells and inhibits protein synthesis [42]. The RTB itself was non-toxic and it could recognize terminal galactose residues and facilitate the interaction between type II RIPs and the cell membrane [43]. According to proteomic data from *P. volubilis*, no RTB proteins were identified, and based on transcriptomic data, 4 transcripts encoding ricin B-like lectin had very low expression levels. Therefore, it can be inferred that the RIPs in *P. volubilis* would not be a serious threat to public safety unless the factors (e.g., biotic or abiotic stress, plant hormones) [22] affect their expression levels in the plants.

The expression patterns of 10 RIP genes in different organs of *P. volubilis* were assessed by RT-qPCR. They were all highly expressed at two developmental stages of *P. volubilis* seeds, among which, six were expressed at the highest levels in seeds at the fast oil accumulation stage. At the initial stage, however, 3 RIP genes were expressed at higher levels. The results of our study corroborated the previous findings obtained by Llediad et al. [44] and Loss-Morais et al. [22] who reported that the expressions of members of the RIP gene family were often tissue-specific and developmental stage-specific. In addition to being specifically and highly expressed in seeds, Unigene0000172 also had higher expression levels in stem and stem apex. Additionally, Unigene0030485 encoding type I RIPs had the highest expression level in roots. Type I RIPs are widely distributed in most organs of many plants, and the contents of these genes vary greatly in different organs of different plants. The toxicity of type I RIPs is generally low, but they have certain antiviral properties since the viral infection in plants promotes the passage of the RIP into the cells to inhibit the replication of the virus. Furthermore, plant RIPs play an important role in defense against various environmental stresses and can activate plant defense systems [45]. There have been many research findings that revealed that the expression of RIPs could be activated by some factors, such as phytohormones, viral infection, development, senescence, and environmental stress [46,47,48]. The expression of Unigene0030485 was higher in the roots of *P. volubilis*; however, it remains to be determined whether it is related to antiviral activity or various environmental stresses.

In addition, the results of co-expression analysis and protein interaction network analysis showed that there has little correlation between oil accumulation and ricin accumulation. Transcriptional regulatory network analysis showed that only 4 ricin B-like genes with low expression levels could be associated with the other 29 genes, and 4 *LACS8* transcripts represented potential targets for 3 RIP genes. LACSs play vital roles in lipid biosynthesis and fatty acid metabolism in plants [49]. However, Zhao et al. [50] reported that disruption and overexpression of *LACS8* did not affect the seed fatty acid content in *Arabidopsis*. These findings also provide evidence that the oil obtained from *P. volubilis* would be safe for human consumption.

## 4. Materials and Methods

### 4.1. Sample Collection

The 3-year-old *P. volubilis* trees introduced from Peru were cultivated at the South China Experimental Nursery (21°30′ N, 111°38′ E, 90 asl), Guangdong, China, under natural conditions. In the study, the strain number of V3, which has excellent oil quality and high yield, was selected as the research material. Based on the dynamic changes of fatty acid accumulation in developing seed [23], we analyzed the transcriptome and proteome data from samples at two developmental stages (the initial stage of seed development, Pv-1, and the fast oil accumulation stage, Pv-2), which were found to be consistent with those reported by Wang et al. [21]. The developing seeds did not start to accumulate large amounts of fatty acids, especially the α-linolenic acid, in Pv-1 and mostly in Pv-2. The mature female flowers were tagged and hand pollinated. Flowers were collected 10 DAP at Pv-1 and 100 DAP at Pv-2. In addition to the seeds (Pv-1 and Pv-2), mature leaves (Pv-ML), unfold young leaves (Pv-YL), pericap (Pv-PC), stem (Pv-ST); stem apex (Pv-SA); stem bark (Pv-SB) and root (Pv-RO) were sampled to analyze the expression patterns of target genes. Each sample was taken from three different plants, and for each stage, three biological replicates were considered.

### 4.2. Transcriptome Analysis

A transcriptome profile was examined at two stages of seeds using RNA sequencing (RNA-seq). Total RNA was extracted using the TRIzol reagent kit (Invitrogen, Carlsbad, CA, USA). The quality of RNA was assessed on an Agilent 2100 Bioanalyzer (Agilent Technologies, Palo Alto, CA, USA) and double-checked using RNase-free agarose gel electrophoresis. The samples with the RNA integrity number (RIN) higher than 8.0 determined by the Agilent 2100 Bioanalyzer were used for the construction of sequencing libraries. The steps involved the enrichment of mRNA by Oligo (dT) beads, RNA fragmentation, synthesis of the second-strand cDNA, size selection, and PCR amplification. The two libraries were then sequenced using Illumina HiSeq^TM^ 4000 by Gene Denovo Biotechnology Co., (Guangzhou, China).

To get high-quality clean reads, the obtained 150 bp paired-end reads were further filtered for quality by FASTP (version 0.18.0, Shenzhen, China) [51]. The quality filtering was performed by removing the reads containing adapters, low-quality reads containing more than 50% of low-quality (*Q*-value ≤ 20) bases and reads containing more than 10% of unknown nucleotides. Due to the absence of a reference genome, de novo transcriptome assembly was carried out using the short-read assembly program Trinity (version 2.8.6, Cambridge, MA, USA) [52]. The transcriptome integrity was assessed using Benchmarking Universal Single-Copy Orthologs (BUSCO, version 3.0.2, Geneva, Switzerland) [53] that incorporated 1440 single-copy orthologous genes as the embryophyte dataset.

The unigenes were annotated by performing BLASTx searches (http://www.ncbi.nlm.nih.gov/BLAST/, accessed on 29 August 2021) with an E-value threshold of ≤1 × 10^−5^ against the non-redundant protein database (Nr) in NCBI (http://www.ncbi.nlm.nih.gov, accessed on 29 August 2021), the SWISS-PROT protein sequence database (http://www.expasy.ch/sprot, accessed on 29 August 2021), the Gene Ontology (GO) database (http://geneontology.org/, accessed on 29 August 2021), the Kyoto Encyclopedia of Genes and Genomes (KEGG) database (http://www.genome.jp/kegg, accessed on 29 August 2021), and the COG/KOG database (http://www.ncbi.nlm.nih.gov/COG, accessed on 29 August 2021). Further analysis of unigene differential expression was carried out between two libraries using DESeq2 [54] and edgeR [55] software programs. The differentially expressed genes (DEGs) were identified based on criteria set as the FDR ≤ 0.05 and the fold change (FC) ≥ 2. Gene ontology (GO) and KEGG pathway enrichment analyses of DEGs were conducted by the hypergeometric test.

### 4.3. Proteomics Analysis

Total protein extraction was determined in three biological replicates of each sample using the cold acetone method. The quality of proteins was evaluated with SDS-PAGE, and the BCA Protein Assay Kit was used to determine the protein concentrations in the supernatant. Around 100 µg of protein for each sample was transferred to a new microcentrifuge tube, and the final volume was adjusted to 100 µL with 8 mol/L urea. Thereafter, 2 µL of 0.5 mol/L TCEP was added and the sample was incubated at 37 °C for 1 h, followed by the addition of 4 µL of 1 mol/L iodoacetamide into samples and incubation for 40 min at room temperature in the dark. Five volumes of −20 °C pre-chilled acetone were added to precipitate the protein extracts at −20 °C overnight. Around 1 mL pre-chilled 90% acetone aqueous solution was used to wash protein precipitate twice, followed by re-dissolving in 100 µL 100 mmol/L TEAB. To digest the proteins at 37 °C overnight, sequencing-grade modified trypsin (Promega, Madison, WI, USA) was added at the ratio of 1: 50 (an enzyme (wt): protein (wt)). C18 ZipTip was used for desalting the peptide mixture, which was then quantified by Pierce™ Quantitative Colorimetric Peptide Assay (23,275) and lyophilized by SpeedVac.

The resultant peptides were labeled by the iTRAQ-8PlexIsobaric Mass Tag Labeling Kit (Thermo Fisher Scientific, Waltham, MA, USA), pooled, and lyophilized in a vacuum concentrator. Subsequently, the peptides were redissolved and separated at high pH. Twelve separated fractions were collected from each sample and identified after drying.

Each collected peptide fraction was re-dissolved and analyzed by online nanospray LC-MS/MS on an Orbitrap Fusion Lumos coupled to the EASY-nLC 1200 system (ThermoFisher Scientific, Waltham, MA, USA). A 2 kV electrospray voltage was set on the inlet of the mass spectrometer. The mass spectrometer was operated in the data-dependent acquisition (DDA) mode and could automatically be switched between MS/MS and MS mode.

DIA raw data were processed and analyzed with Spectronaut X (Biognosys AG, Zurich, Switzerland) under default settings. The retention time prediction type was set to dynamic iRT. Data extraction was determined by Spectronaut X based on the extensive mass calibration. The Spectronaut Pulsar X was used to dynamically determine the ideal extraction window based on iRT calibration and gradient stability. The *Q*-value (FDR) cutoff at the precursor and protein levels was set to 1%. Decoy generation was set to mutate, which was similar to scrambled but only applied a random number of AA position swamps (min = 2, max = length/2). All screened precursors which were passed through filters were used for quantification. The major group quantities were calculated using the average top 3 filtered peptides, which passed the 1% *Q*-value cutoff. After Student’s *t*-test, the Benjamini and Hochberg method was applied, and DAPs were filtered with the fold change (FC) > 1.2 and FDR < 0.05.

The GO, KEGG, and COG/KOG databases were used to annotate proteins and predict their functions. The significantly enriched GO terms and KEGG pathways of DAPs with *Q*-value ≤ 0.05 were identified. MASCOT [56] was used to analyze the DAPs with the FC > 1.2 and *p* < 0.05 at two different developmental stages of *P. volubilis* seeds.

### 4.4. mRNA and Protein Association Analysis

A qualitative correlation was established between the genes that were regulated in a similar direction by RNA and protein expression [57], and then the Venn diagram was plotted [58]. For the analysis of the correlation between transcriptome and proteome data, changes in protein levels complementary to the changes in the corresponding transcripts were investigated, and the correlations between DEGs and DAPs were depicted by drawing a nine-quadrant map in R (version 3.5.1, Boston, MA, USA). The correlation analysis of the GO annotation and KEGG pathway was performed between the transcriptome and proteome data, and the similarities and differences between the gene function and metabolic pathway in two groups of data were compared.

### 4.5. Bioinformatics Analysis of RIP Genes

Based on the NR and SwissProt annotation results, 21 transcripts of RIPs were screened. The Open Reading Frame Finder (ORF Finder) online program at NCBI (https://www.ncbi.nlm.nih.gov/orffinder, accessed on 2 March 2022) was used to determine the ORFs of these transcripts. The Conserved Domain Database (CDD) (https://www.ncbi.nlm.nih.gov/Structure/cdd/wrpsb.cgi, accessed on 2 March 2022) was used to identify conserved domains in protein sequences [59]. The ProtScale server (http://www.web.expasy.org/protscale, accessed on 2 March 2022) was used for the analysis of basic physicochemical properties of amino acids, including the number of amino acids, theoretical isoelectric point (pI), instability index (II), aliphatic index, and grand average of hydropathicity (GRAVY) [60], and the TMHMM tool (https://services.healthtech.dtu.dk/service.php?TMHMM-2.0, accessed on 2 March 2022) was used to analyze the transmembrane protein structure. A total of 23 amino acid sequences of other plant species were selected for phylogenetic tree analysis. The IQ-TREE 2.2.0 software [61] was used to construct the phylogenetic tree using the maximum likelihood (ML) method with 1000 bootstrap replicates. All amino acid sequences were aligned using the MUSCLE method.

### 4.6. Quantitative Reverse Transcription PCR (RT-qPCR)

The relative expression of the 10 selected genes was characterized using total RNA extracted at two developmental stages of *P. volubilis* seeds. Real-time quantitative polymerase chain reaction (RT-qPCR) was performed with all RNA samples using gene-specific oligonucleotide primers (Table S13), HiScript II Q RT SuperMix (Vazyme, Nanjing, China) with iQ SYBR Green Supermix (TaKaRa Bio, Beijing, China), and the Thermo Scientific PikoReal 96 Real-Time PCR System (Thermo Fisher, Waltham, MA, USA). All obtained values were normalized to the *Actin* gene (Unigene0042747) and then standardized to control conditions. Error bars represent the standard error of the mean (SEM), and the Student’s *t*-test, a statistical significance test, was used to compare the means of groups, with *p* < 0.05 considered to be statistically significant. After performing RT-qPCR, *Ct* values were obtained using the ABI StepOnePlus^TM^ software. The 2^–ΔΔCt^ method [62] was used to calculate the expression level of mRNA and FC values. Three biological replicates and three technical replicates were considered for each treatment in all experiments.

## 5. Conclusions

Numerous studies claimed that the plant RIPs have been connected to defense by the antifungal [63], antibacterial [64], antiviral [65], and anti-pest agent activities [43,66]. So, RIP has the potential to be widely used as biopesticides, and it has been extensively studied for its insecticidal activity. Due to its potential to inhibit protein biosynthesis, RIP can be used as a good target gene in plant genetic engineering for disease resistance. However, the related functions of RIP in *P. volubilis* need to be further studied. The screened transcripts in this study, including four transcripts (Unigene0000172, Unigene0000207, Unigene0000208, and Unigene0000209) encoding ricin-like proteins and Unigene0012386 encoding type I RIPs were highly expressed in Pv-2, and thus, they will be the focus of further research.

## Data Availability

The raw data of sequenced transcriptome have been deposited to the Sequence Read Archive (SRA) at NCBI with the accession number of SRP387519 (http://www.ncbi.nlm.nih.gov/sra, accessed on 22 July 2022). The mass spectrometry data have been submitted into the iProX with the accession number of IPX0004771000 (http://www.ncbi.nlm.nih.gov/sra, accessed on 25 July 2022).

## Supplementary Materials

The following supporting information can be downloaded at: https://www.mdpi.com/article/10.3390/ijms23179562/s1.
