# Supplementary material for: Integrated Transcriptome and Proteome Analysis Provides Insight into the Ribosome Inactivating Proteins in Plukenetia volubilis Seeds
Source: Int J Mol Sci. 2022 Aug 24;23(17):9562. doi: 10.3390/ijms23179562 (PMC9455912; doi:10.3390/ijms23179562)
Supplement: Supplementary file 1 [file ijms-23-09562-s001.zip › Supplementary materials/Supplementary Figures.pdf]

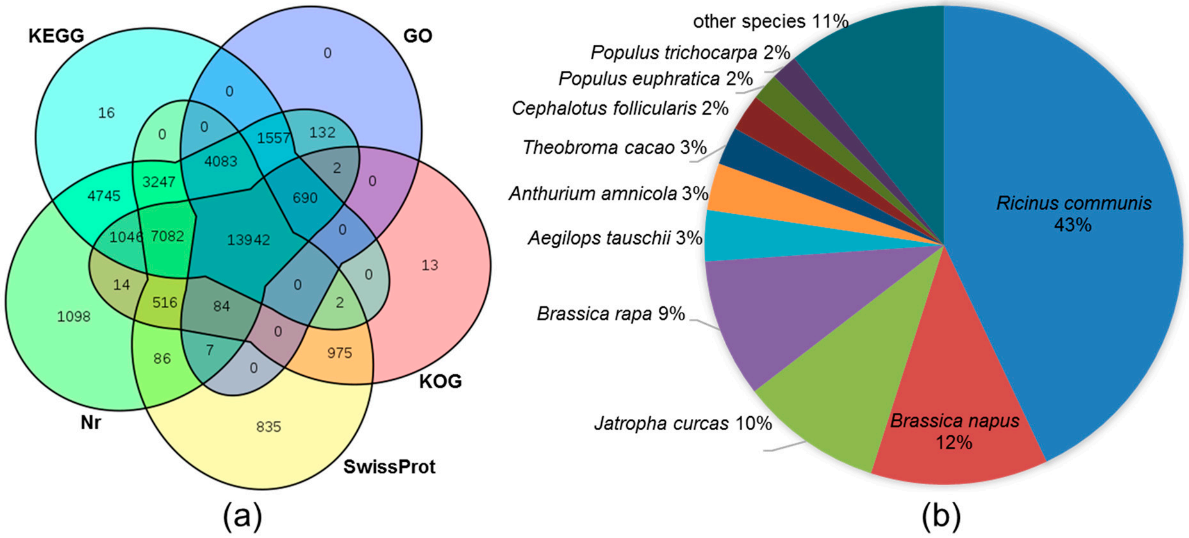

Figure S1. (a) Venn diagram of number of transcripts annotated by BLASTx against GO, KEGG, Nr, SwissProt, and KOG databases. **(b)** The numbers of transcripts matching the 10 top species using BLASTx in the Nr database.

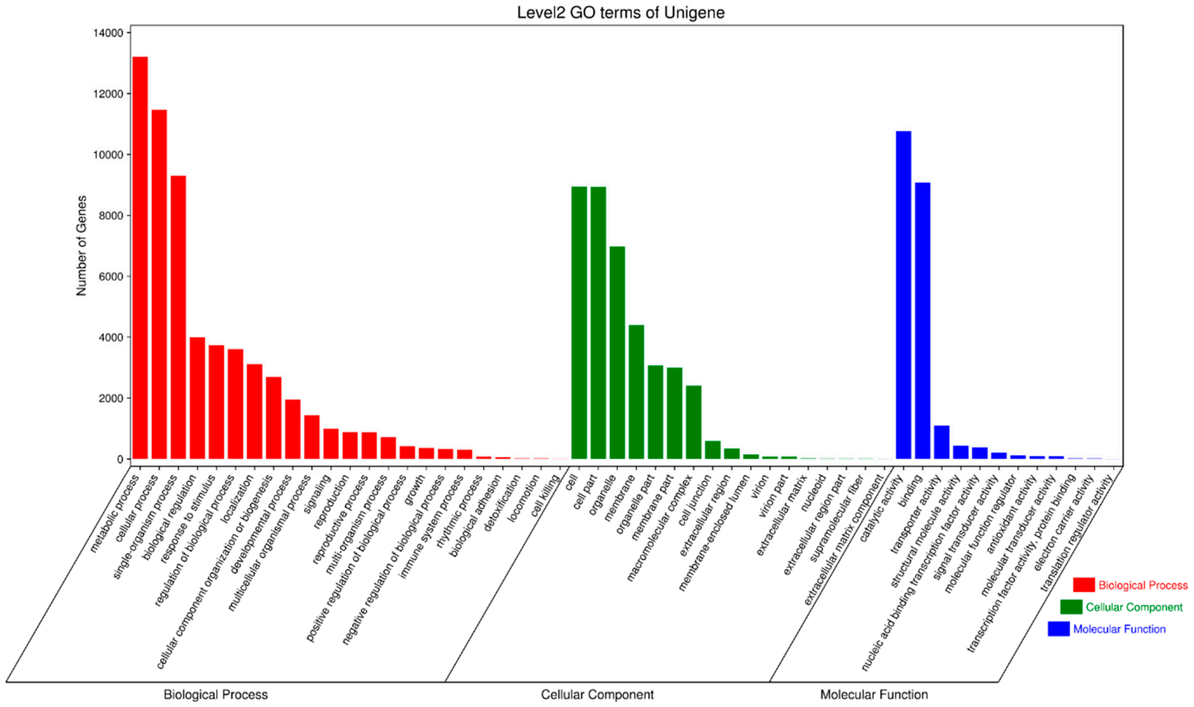

Figure S2. GO categories for transcripts in the transcriptome. The y-axis represents the number of transcripts in a category.

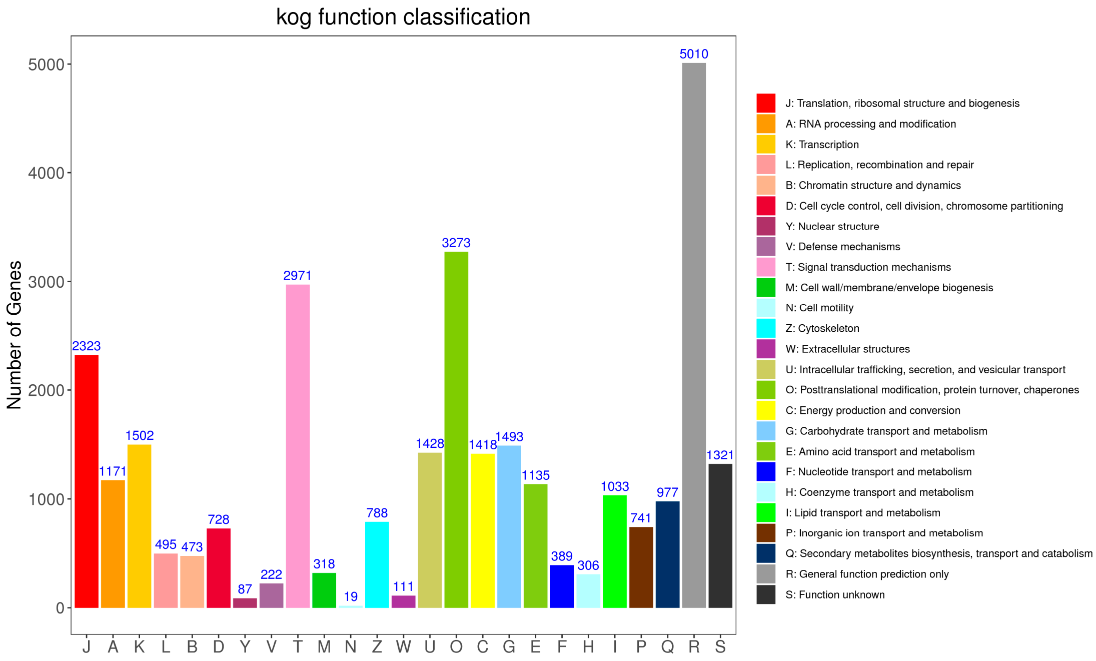

Figure S3. KOG functional classification for the transcriptome sequences.

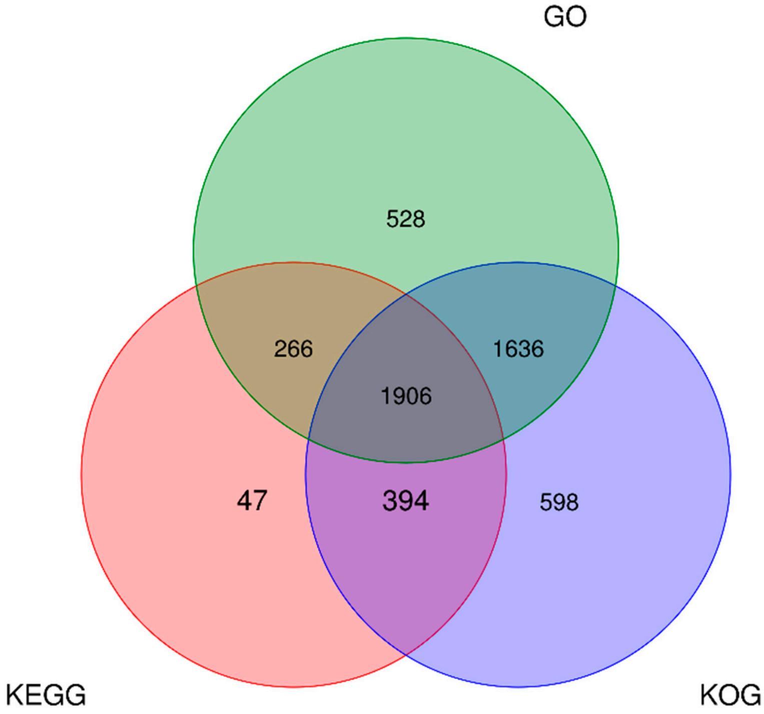

Figure S4. Venn diagram of identified proteins numbers annotated by BLASTx against GO, KEGG and KOG databases.

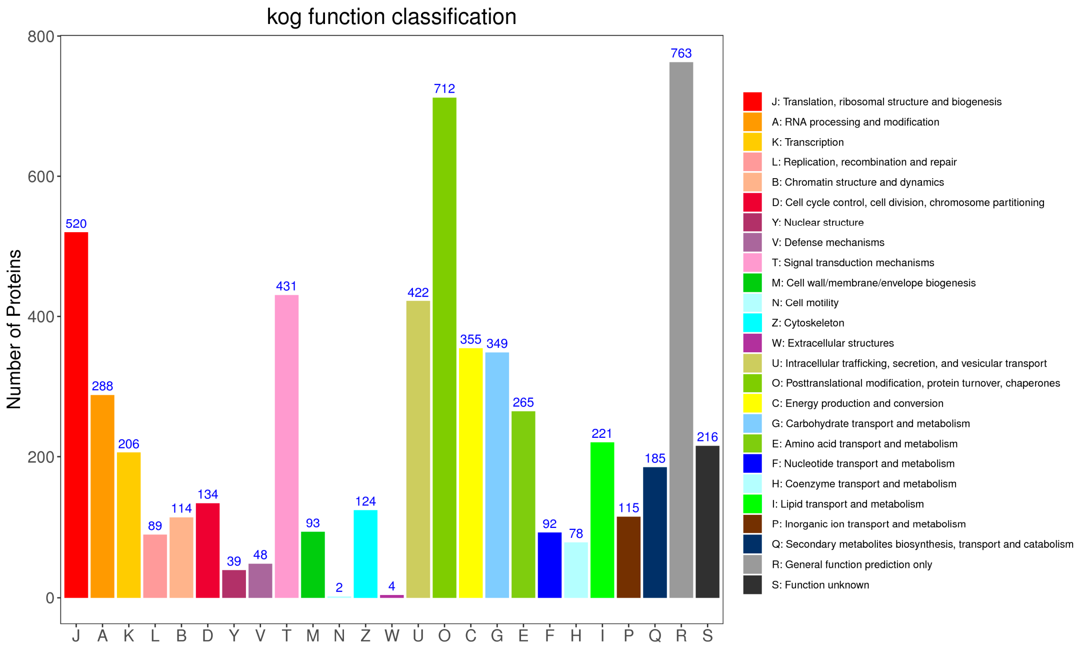

Figure S5. KOG functional classification of all identified proteins.

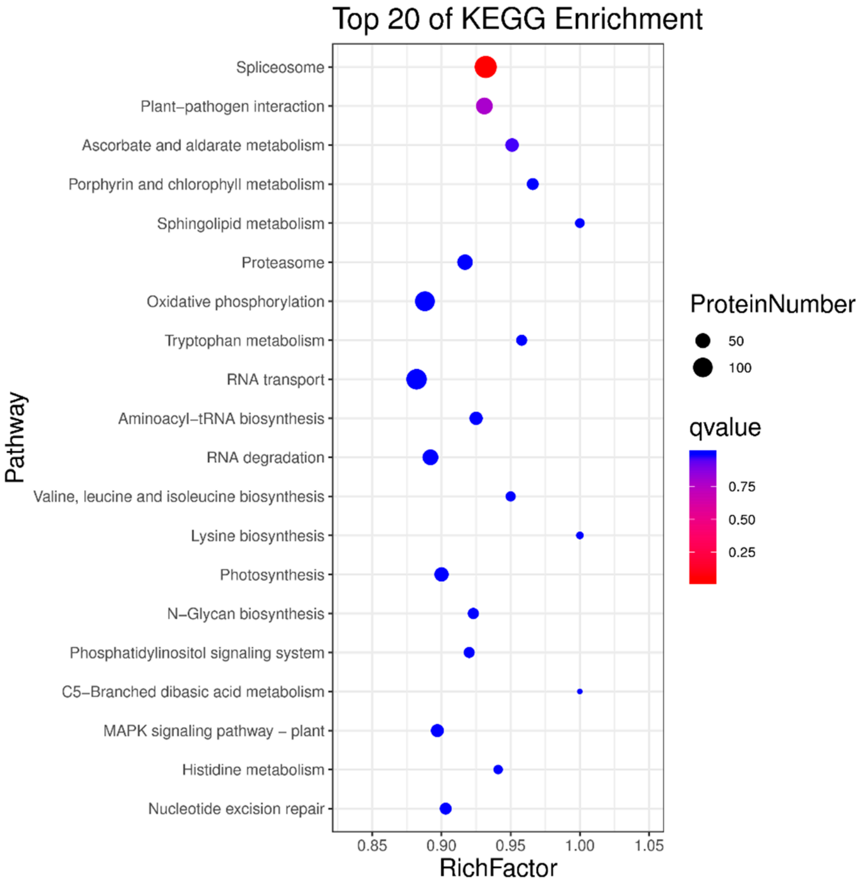

Figure S6. Top 20 KEGG pathways enriched by unique differentially abundant proteins between the Pv-1 and Pv-2.

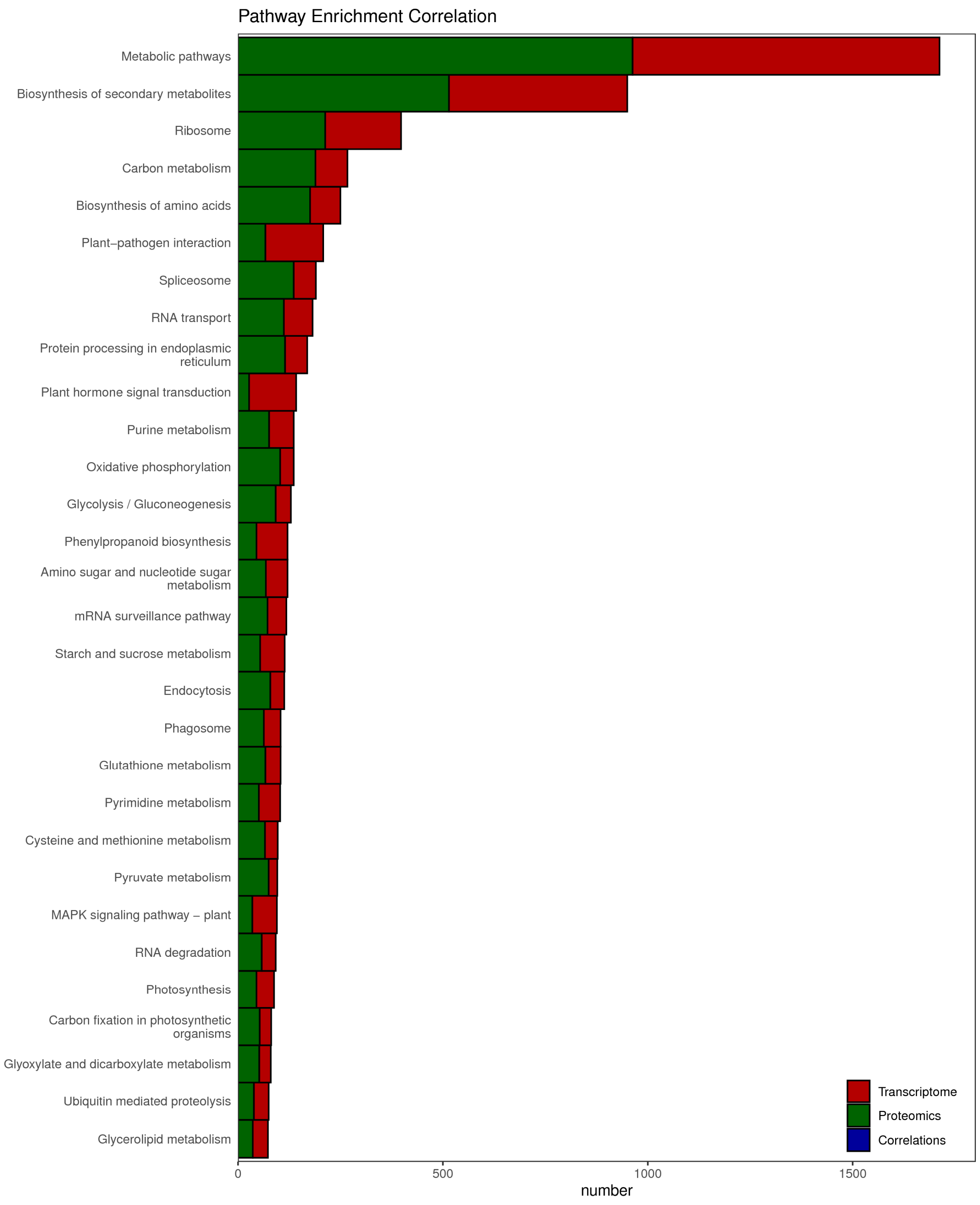

Figure S7. KEGG pathway enrichment of DEGs/DAPs. Red bars represent DEGs in the transcriptome; green bars represent DAPs in the proteome; blue bars represent the number of both DEGs and DAPs.

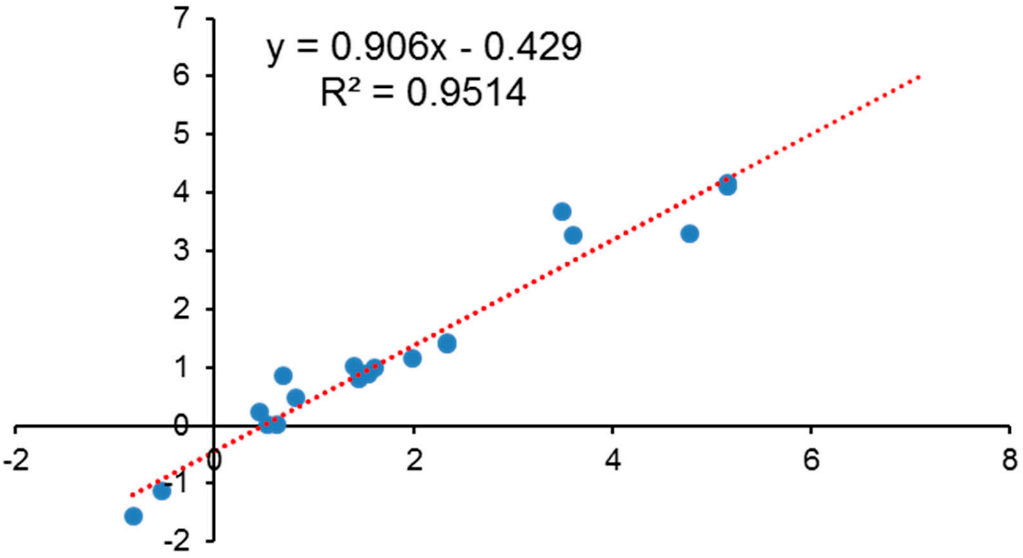

Figure S8. The correlation between qRT-PCR and the transcriptome data, as shown by values of log2(RPKM ratios) obtained by RNA-seq (x-axis), plotted against the values of log2(relative expression ratios) obtained by qRT-PCR (y-axis) for the ten RIP genes. Each sample was analyzed in three biological replicates.
